# Why tRNA acquisition could be relevant to bacteriophages?

**DOI:** 10.1111/1751-7915.14464

**Published:** 2024-04-18

**Authors:** Carlos O. Lomeli‐Ortega, José Luis Balcázar

**Affiliations:** ^1^ Catalan Institute for Water Research (ICRA‐CERCA) Girona Spain; ^2^ University of Girona Girona Spain

## Abstract

In this opinion, we discuss the role of tRNAs in phage biology and their importance in DNA replication and phage‐host interactions. Phages are a diverse group of obligate bacterial viruses that possess genomes with a wide range of sizes. Among them, we find phages with few genes that depend entirely on their host's translational machinery for replication. However, some phages carry genes for all replication steps and even contain genes for their own translational synthesis. In these cases, the integration of tRNA genes in their genomes is not completely understood, generating different theories about their presence and function during the replication cycle. Although different studies have attempted to elucidate their role, additional studies are needed to clarify the presence and significance of tRNA genes in phages. Moreover, we highlight the importance of tRNA genes in phages from both ecological and therapeutic perspectives.

## Graphical abstract

We highlight the role and advantages of tRNA genes in phages, and a perspective about the use of phages with tRNA genes in biotechnological applications to contribute to new perspectives among the scientific community.

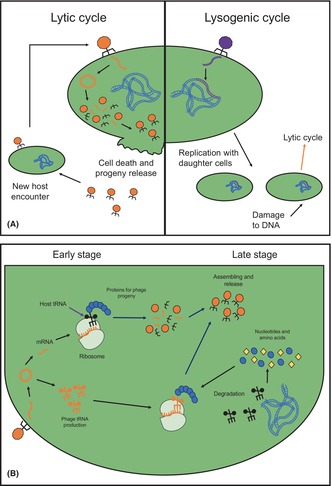

Bacteriophages, or simply phages, are viruses that specifically infect bacteria and use them as microbioreactors to reproduce, ultimately leading to the death of the hosts at the end of the lytic cycle (Figure 1A). Additionally, phages can survive inside their host, particularly under adverse environmental conditions, thereby enhancing host metabolic capacity (Hatfull, 2008; Figure 1A). This latter interaction is known as lysogeny, where lysogenic or temperate phages can carry a diverse set of genes from previous infections, including antibiotic resistance and xenobiotic degradation genes (Sun et al., 2023; Zheng et al., 2022). These types of genes are known as auxiliary metabolic genes (AMGs). Unlike essential components of the phage replication cycle, such as structural proteins, ATP production, chaperone proteins and the endolysin‐holin complex, AMGs are not necessary for phage replication. However, they can be expressed to enhance or support host metabolism (Pfeifer et al., 2022). While AMGs are more frequently found in lysogenic phages due to their lifestyle, they can also occur in lytic phages, despite their simpler genetic machinery. Lytic phages infect their hosts and utilize the host's metabolic machinery to produce proteins necessary for viral structure and genetic material for new progeny. After assembly, viable progeny is released upon host disintegration. This new progeny will repeat the same cycle if a susceptible host is available. Due to this lifecycle, the function of AMGs in lytic phages remains unclear, primarily because a diverse set of genes with unknown functions is found in double‐stranded DNA (dsDNA) lytic phages. Among them, the presence of tRNA genes catches our attention, because tRNAs, along with other translation‐related genes used for phage replication, belong to the host translational machinery (Kauffman et al., 2018; Yang et al., 2021).

**FIGURE 1:**
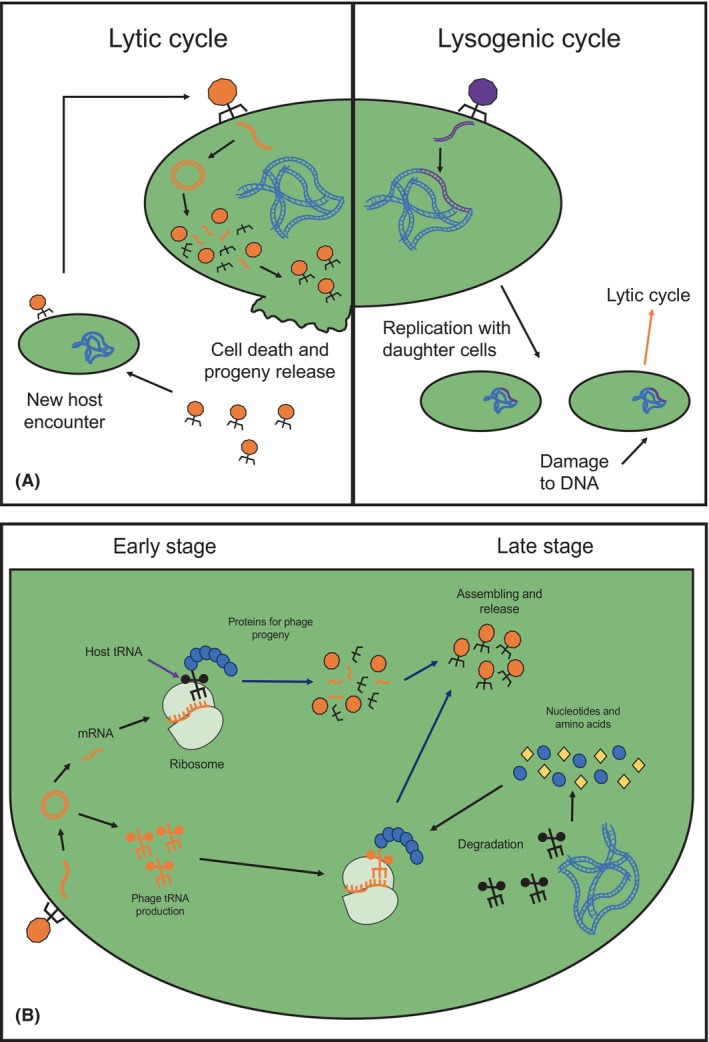
(A) Lytic and lysogenic life cycles of phages. During the lytic cycle, phages inject their genetic material into bacteria, replicate within the host and ultimately lyse the host cells, releasing new viral particles. In contrast, during the lysogenic cycle, phages can integrate their genomes into the host chromosome as prophage, remaining dormant until triggered to enter the lytic cycle. (B) During the lytic cycle, the phage's genetic material expresses mRNA to synthesize proteins involved in the conformation and assembly of new progeny and accumulates their own tRNAs for use in a late stage. When genetic material is depleted, host DNA, tRNAs and other components will be degraded, and to compensate for this, phage tRNAs will be used to continue the production of proteins in order to finish the lytic cycle.

However, tRNA genes have been found inside the genomes of several phages without any clear reason for their presence or function (Bailly‐Bechet et al., 2007). Several theories have been proposed in recent years to explain their functions. For instance, the presence of tRNA genes may be related to erroneous prophage excision or random DNA uptake during new progeny assembly in the lytic cycle (Pfeifer et al., 2022). However, these cases cannot fully explain why lytic phages carry and maintain tRNA genes (Yang et al., 2021). In fact, there is a notable difference in the presence of tRNA genes between lytic and lysogenic phages, as these genes are mainly found in lytic phages rather than lysogenic ones. A plausible explanation is that their presence is a consequence of the replication cycle needs for large phage genomes (genomes larger than 200 kb; Al‐Shayeb et al., 2020). In this scenario, larger phages degrade the host genome to obtain more nucleotides, thereby increasing their burst size and compensating for the decrease in host translation machinery by using their own machinery to extend the replication time (Figure 1B). In the case of the random DNA uptake theory, a gene should confer some benefit for its presence to be conserved, such as an increase in fitness, antibiotic resistance and anti‐phage infection mechanisms, among others. However, in the case of tRNA genes, their specific benefit is not yet completely understood. Considering these points, one theory suggests that the expression of tRNA helps phages during the replication cycle by compensating for differences in codon usage between the phages and their hosts (Bailly‐Bechet et al., 2007; Yang et al., 2021). In this theory, phages use codons that are not preferential for the host because their own tRNA genes will compensate for these differences. However, this is not a strict rule because some phages have most of their genes, or even their entire genome, biased towards host‐preferred codons to enhance their replication (Carbone, 2008). Therefore, the presence of tRNAs could be more closely related to phage adaptation to increase their broad host range (Miller et al., 2003), considering that each host may have a dominant codon composition (Carbone, 2008).

Although the presence of phage tRNAs helps with translation, this dominance is not consistent throughout all stages of replication because it is more common to observe the expression of host tRNAs over phage ones in the early phases (Figure 1B). One explanation could be that early phage mRNAs rely on host tRNAs while phage tRNAs are being transcribed and processed. In contrast, late mRNAs become more dependent on phage tRNAs as host tRNAs may be degraded at a later stage (Yang et al., 2021). Although the theory about the compensating role of tRNAs is not completely correct because phage tRNA genes do not participate during the complete replication cycle to compensate for differences in codon usage bias, phage tRNAs act as a support component for translation. Moreover, codon usage bias is more pronounced in late phage genes than in early genes due to the degradation of host components. Another plausible explanation is that phage‐encoded tRNAs are involved in evading bacterial defence, enabling them to counteract the tRNA‐depleting strategies of the host using enzymes such as VapC, PrrC, colicin D and colicin E5 to defend against phage infection (Azam et al., 2023; van den Berg et al., 2023). These factors could explain why multiple types of tRNAs are simultaneously present in phage genomes and why such phages exhibit a broad host range (Azam et al., 2023). Previous studies have also demonstrated that many genomes of crAss‐like phages encode multiple tRNAs including putative suppressors, which can be perceived as an anti‐defence strategy to disrupt the production of host proteins, including those involved in defence, through stop‐codon readthrough, while ensuring the accurate translation of phage proteins (Borges et al., 2022; Yutin et al., 2021). In addition to their potential to enhance translational efficiencies during the lytic cycle or counteract host defences that degrade host tRNAs, it has recently been proposed that phage‐encoded tRNAs play key roles in the establishment of lysogeny in some temperate phages (Guerrero‐Bustamante & Hatfull, 2024). These situations demonstrate that the selection and conservation of tRNA genes in genomes have important implications for phage biology and ecology.

Phages have immense potential to serve as therapeutic agents against bacterial infection or to control undesirable bacteria in several sectors including industry, agriculture and human health due to their selectivity for bacterial hosts and their ability to destroy bacterial host cells at the end of the lytic cycle. Given this, the presence of tRNA genes can confer an advantage to phages over those lacking them. In hosts with compressed genetic codes or lacking tRNA (Nyerges et al., 2023), phages with tRNA can use their own genes to compensate for the missing ones and evade anti‐phage defences. This enables phages to initiate the replication cycle without difficulty, thereby increasing their host range (Delesalle et al., 2016). Also, the presence of tRNA genes in larger phages allows them to cover longer periods to synthesize their progeny because of both the larger progeny size and the unavailability of host translational machinery due to degradation in late infection (Al‐Shayeb et al., 2020; Nyerges et al., 2023).

In conclusion, given that the presence of tRNAs in phages has not been completely understood, there are several knowledge gaps about their function(s) and the advantages that they provide. However, it has been observed that their presence favours the replication cycle, probably providing an advantage for expanding their host range and allowing large phages to increase their progeny. This would allow the selection of phages larger than their predecessors. Although laboratory models cannot exemplify what happens in nature, they are a good tool for understanding the basic aspects of tRNAs, thereby generating information about their role during lytic and lysogenic cycles, the mechanism(s) of acquisition and selection, as well as determining if an increase in tRNA genes allows a higher host availability. This kind of information could demonstrate the potential of phages with tRNA genes in biotechnological and therapeutic applications, and even allow the genetic modification of phages without tRNA genes to increase their infectivity and host range.

## AUTHOR CONTRIBUTIONS

**Carlos O. Lomeli‐Ortega:** Conceptualization; writing – original draft; writing – review and editing. **José Luis Balcázar:** Conceptualization; funding acquisition; writing – original draft; writing – review and editing.

## CONFLICT OF INTEREST STATEMENT

The authors declare no conflict of interest.
